# Abstract of a Memoir on the Febrifuge Principle of Cinchona

**Published:** 1804-03-01

**Authors:** 


### *Abstract of a Memoir on the Febrifuge Principle of Cinchona, by* Cit. Seguin.

THE object proposed to himself by the author in the task he undertook was, to point out the means of knowing with certainty the true febrifuge principle of cinchona; to distinguish the species that contain it from those that do not; and, lastly, to appreciate its quantity and quality.

Hitherto the sight and taste have been the only tests of the prsumable qualities of the Peruvian bark of the shops; but as these have no precise standard, and are inapplicable to powdered bark, they very imperfectly indicate the presence of the febrifuge principle. It was of importance, therefore, to substitute to these means, little better than illusory, others not only capable of calculation, but likewise invariable. Chemical re-agents alone can answer these ends.

In consequence, Cit. Seguin began by isolating the respective properties of all medicinal substances, and he examined the action they exert on all other chemical substances.

These researches led him to develope very decisive characteristics in the febrifuge principle of cinchona, which place it in a perfectly distinct class. The following are its characters.

It precipitates the solution of tan, but not the solutions of gelatine and sulphate of iron.

When cinchona has not all these characters, it is a proof that it is mixed with something else, or that it does not contain the febrifuge principle.

 The

---

The author has subjected to this analysis all the known species of cinchona, found among all the druggists and apothecaries of Paris and Versailles, and constantly obtained the same results.

Unfortunately, these researches have shown, that but an infinitely small quantity of good, unmixed cinchona, is to be procured in the shops; the greater part being either destitute of the febrifuge principle, or mixed, or of a very inferior quality, though containing no mixture.

These results are of so much the greater importance, because the effects of different kinds of cinchona, in fevers, are only in proportion to the greater or less quantity of the febrifuge principle they contain; and those which contain none, as well as all the substances that may be mixed with them, are more or less injurious to the system.

The experiments of Cit. Seguin on the febrifuge principle of cinchona, having convinced him that most of the bark found in the shops was injurious or inefficacious, because it was spoiled by keeping, adulterated by mixture, or deprived of the febrifuge principle; he has endeavoured to obtain a febrifuge principle always the same, more efficacious, more certain in its effects, more capable of assimilation with our sysem, and so cheap, that there could be no temptation to adulterate it.

To attain this important object, the author has inquired what the true cause of fevers, as of their effects, is; what the nature of the febrifuge principle of cinchona, and what its action on our system. He has subjected to the action of the re-agents pointed out for the febrifuge principle of cinchona, all chemical and medicinal substances; and assured himself, whether such of these substances, as might contain the febrifuge principle, did not contain, at the same time, other substances prejudicial to the animal economy. Lastly, he had to cure fevers by the help of these remedies, and then confirm this theory by repeated experiments. Such is the course Cit. Seguin has pursued.

The new febrifuge principle, which he proposes to substitute instead of cinchona, because it unites all the advantages of the bark, without any of its inconveniences, is gelatine in its pure state.

Considered in a medical, economical, and political view, gelatine promises much greater advantages than bark, in its application to the cure of fevers. It occasions no irritation; procures quiet sleep and gentle perspiration; keeps the belly open, without producing cholic or nausea; has no unpleasant flavour; restores the strength, and is digested ed even by the weakest stomach, that would reject the bark as soon as administered.

On the other hand, cinchona irritates the system, disturbs the sleep, has a disagreeable taste, frequently occasions costiveness, and is very indigestible.

In an economical view, there is still greater difference between cinchona and gelatine; the price of the latter being to that of the former, at most, as one to thirty-two.

Lastly, gelatine is indigenous, cinchona is not; and the purchase of the latter requires us to send abroad a very considerable sum of money, which might be kept at home by adopting the use of gelatine.

To this memoir the author has subjoined thirty-seven cases, in which he performed a cure with gelatine, under the eyes of some respectable physicians, and he has desired a Committee to be appointed, to repeat his experiments, and report upon them.

Accordingly, Citizen Portal, Desessarts, Hallé, Fourcroy, Berthollet, and Deyeux, have been nominated for this purpose. Their experiments are made at the School of Medicine, in a room exclusively appropriated to these inquiries; already a great number of patients have been cured; and the Committee will soon make their first report on these cases.

